# Prevalence of antimalarial drug resistance molecular markers in Makenene, central Cameroon

**DOI:** 10.1016/j.ijpddr.2026.100633

**Published:** 2026-01-16

**Authors:** Nelly Armanda Kala-Chouakeu, Joel Djoufounna, Nicolas Benoit, Timoléon Tchuinkam, Océane Delandre, Lionel Almeras, Roland Bamou, Christophe Antonio-Nkondjio, Marylin Madamet, Bruno Pradines

**Affiliations:** a Vector Borne Diseases Laboratory of the Research Unit of Biology and Applied Ecology (VBID-RUBAE), Department of Animal Biology, Faculty of Science of the University of Dschang, Cameroon; b Organisation de Coordination pour la Lutte Contre les Endémies en Afrique Centrale (OCEAC), Yaoundé, Cameroon; c Unité Parasitologie et Entomologie, Département Risques Vectoriels, Institut de Recherche Biomédicale des Armées, Marseille, France; d Aix-Marseille Univ, SSA, AP-HM, RITMES, Marseille, France; e IHU Méditerranée Infection, Marseille, France; f Centre National de référence du paludisme, Marseille, France; g Laboratory of Malaria and Vector Research, NIAID, NIH, Rockville, MD, USA

**Keywords:** Malaria, *Plasmodium falciparum*, In vitro resistance, Antimalarial drugs, Molecular markers, Cameroon

## Abstract

**Background:**

Antimalarial drug resistance remains a significant challenge in the fight against malaria in Cameroon. Given the high prevalence of malaria in Makenene in central Cameroon and the limited knowledge of drug resistance profiles in the area, the prevalence of *Plasmodium falciparum* drug resistance genetic markers was assessed.

**Methods:**

185 samples from asymptomatic individuals with *P. falciparum* parasitaemia collected between September and October 2021 were sequenced for the *Pfdhfr*, *Pfdhps*, *Pfcrt*, *Pfmdr1,* and *PfK13* genes.

**Results:**

One hundred percent of the samples harboured parasites with triple mutant *Pfdhfr***I**_51_**R**_59_**N**_108_I_164_ (**IRN**I), associated with high level of resistance to pyrimethamine. The septuple mutant *Pfdhfr***IRN**I and *Pfdhps***A**_436_**G**_437_K_540_**G**_581_**S**_613_ (**AG**K**GS**), associated with resistance to sulfadoxine-pyrimethamine, was detected in 62.0 % of the isolates. The new *Pfdhps* I431V mutation was observed in 18.8 %. The octuple mutant **IRN**I + **VAG**K**GS** haplotype, overrepresented in pregnant women taking intermittent preventive treatment with sulfadoxine-pyrimethamine, was found in 17.4 %. The *Pfdhps* K540E mutation, linked to “super” resistance to sulfadoxine, was only detected in 1.9 %. The *Pfcrt* C_72_V_73_**I**_74_**E**_75_**T**_76_ haplotype, implicated in chloroquine resistance, was absent in Makenene. The *Pfmdr1* N_86_**F**_184_ haplotype, selected in parasites with a recrudescence in patients treated with artemether-lumefantrine, was found in 73.2 %. No isolate harboured the **Y**_86_Y_184_ haplotype, selected in parasites with recrudescence in patient treated with dihydroartemisinin-piperaquine. Moreover, no mutation associated with artemisinin partial resistance was detected in *PfK13*.

**Conclusions:**

The in-depth analysis of genetic mutations associated with antimalarials resistance in this study, notably those with a high prevalence of mutations on the *Pfdhfr* and *Pfdhps* genes, highlights the immediate need for proactive strategies to combat resistance in Makenene. Continuous monitoring, including molecular and in vivo surveillance is crucial to uphold the effectiveness of current treatments and, more particularly, artemisinin-based combination therapies, and to enable better decision-making on effective treatment policy in Cameroon and in Africa as a whole.

## Highlights

• 100 % triple mutant *Pfdhfr* **IRN**I, associated with resistance to pyrimethamine.
• 62.0 % of septuple mutant **IRN**I and **AG**K**GS** (resistance to sulfadoxine-pyrimethamine).
• The new *Pfdhps* I431V mutation was observed in 18.8 %.
• 73.2 % of *Pfmdr1* N**F** haplotype, selected in artemether-lumefantrine resistance.
• No mutation associated with artemisinin partial resistance.

## Graphical abstract

**Figure:**
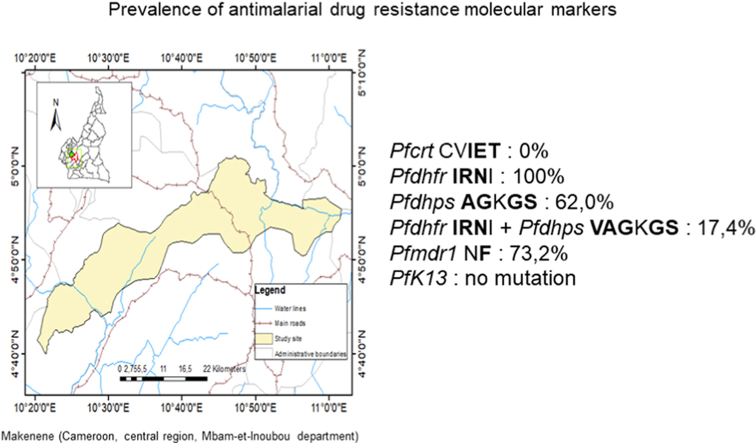

## 1 Introduction

Malaria remains a significant global public health issue in tropical and sub-tropical areas of the world. The highest burden of the disease is in Africa, which is home to up to 94 % of reported cases. Although this disease mainly affects younger children and pregnant women, more than 282 million cases and 610,000 deaths were reported worldwide in 2024, surpassing the estimates of the pre-COVID-19 period (WHO, 2025). Estimated cases of malaria increased by 513,000 (from 7.343 million to 7.856 million) between 2023 and 2024 in Cameroon.

Malaria transmission is stratified in Cameroon (hyperendemic malaria stratum with seasonal transmission inclined to cyclic outbreaks) and the entire population is at risk (Antonio-Nkondjio et al., 2019). To prevent the diseases, the health authorities adopt strategies including vector control and intermittent preventive treatment with sulfadoxine-pyrimethamine (SP) for pregnant women and seasonal malaria chemoprevention with a combination of sulfadoxine-pyrimethamine and amodiaquine (SPAQ) for children aged between 3 and 59 months in the northern regions (WHO, 2013; WHO, 2023). These interventions aim to reduce the burden of malaria and protect the most vulnerable populations from infection and its associated complications.

Prior to 2004 in Cameroon, chloroquine (CQ) and SP were the first-line and second-line antimalarial drugs for the treatment of uncomplicated falciparum malaria (Basco et al., 2006; Sayang et al., 2009; Menard et al., 2012). However, due to the rapid development and spread of malarial resistance to CQ and SP, which could exceed 80 %, including in vivo resistance (Mulder et al., 1992; Agnamey et al., 1995; Ringwald et al., 2000; Basco et al., 2006; Tahar and Basco, 2007a; Mbacham et al., 2010) and the high prevalence of in vitro resistant parasites (Basco, 2002, 2003; Basco and Ringwald, 2007; Tahar and Basco, 2007b) as well as harbouring molecular markers involved in resistance (Basco, 2002; Tahar and Basco, 2006, 2007a; Menard et al., 2012; Apinjoh et al., 2017; Wang et al., 2022; Mbacham et al., 2023; Nana et al., 2023), concerns were raised regarding the effectiveness of these treatments. However, chloroquine-susceptible *P. falciparum* parasites re-emerged around a decade a decade ago (Gharbi et al., 2013; Ndam et al., 2017; Moyeh et al., 2018; Foguim et al., 2020; Wang et al., 2020; L’Espiscopia et al., 2021; Tuedom et al., 2021; Moyeh et al., 2022; Sarah-Matio et al., 2022; Niba et al., 2023; Xu et al., 2023). To address this problem of resistance, since 2004 the treatment of uncomplicated *P. falciparum* malaria has relied on artemisinin-based combination therapies (ACTs), which have proven to be highly effective. The combination of artesunate and amodiaquine (ASAQ) was implemented in Cameroon in 2004 and artemether-lumefantrine (AL) was introduced in 2006 (Achonduh et al., 2014; Antonio-Nkondjio et al., 2019; Youdom et al., 2019). Following the revision of treatment guidelines in 2019, the treatment of uncomplicated *P. falciparum* malaria in Cameroon relies currently on the use of three ACT as first-line therapy, namely artemether-lumefantrine, artesunate-amodiaquine, and dihydroartemisinin-piperaquine (DHPQ) (Programme National de Lutte contre le Paludisme, 2019; Programme National de Lutte contre le Paludisme, 2024). However, in the North and Far North regions, the first-line treatment was reduced to two drugs (AL and DHPQ), while ASAQ was contraindicated due to the implementation of seasonal malaria chemoprevention (SMC), which uses a combination already containing amodiaquine, specifically sulfadoxinepyrimethamine-amodiaquine (SPAQ). In Makenene, the treatment of uncomplicated malaria relies on the use of AL as first-line treatment.

The first-line treatment of severe malaria in Cameroon has been managed with injectable artesunate, the second-line with injectable artemether and third-line with injectable quinine. However, in Makenene, injectable quinine is currently used as first-line treatment.In Cameroon, chemoprevention currently comprises three components: (i) intermittent preventive treatment of malaria in pregnant women (IPTp) with administration of at least three doses of SP starting at 13 weeks of pregnancy on a monthly way until delivery, (ii) intermittent preventive treatment of malaria in infants under 2 years of age (IPTi) or perennial malaria chemoprevention (PMC) with administration of five doses of SP during vaccination visits, and (iii) seasonal malaria chemoprevention (SMC) with the administration of four doses of SPAQ to children aged 3–59 months during high malaria transmission seasons, particularly in the North Region and Far North Region.

No in vivo resistance to artemisinin derivatives in Cameroon has been reported to date, despite certain more or less well-documented clinical failures (Nji et al., 2015; Apinjoh et al., 2016; Menard et al., 2016; Mairet-Khedim et al., 2021; Niba et al., 2022). No mutations known to be associated with parasite clearance delays or in vivo resistance to artemisinin have been reported in Cameroon (Ménard et al., 2016; Apinjoh et al., 2017; Djaman et al., 2017; Eboumbou Moukoko et al., 2019; Wang et al., 2020; L’Espiscopia et al., 2021; Nkemngo et al., 2022).

Resistance is associated with various genetic markers in malaria parasites, including *Pfcrt* for CQ resistance (Fidock et al., 2000), *Pfdhfr* for pyrimethamine resistance (Zolg et al., 1989), *Pfdhps* for sulfadoxine resistance (Wang et al., 1997), *Pfk13* for artemisinin resistance (Ariey et al., 2014), and *Pfmdr1* for resistance to multiple antimalarials such as chloroquine, amodiaquine, mefloquine, quinine, and halofantrine (Price et al., 1999; Reed et al., 2000; Wurtz et al., 2014; Pradines et al., 2020).

It should be noted that more than 25 % of the studies evaluated the prevalence of almost all the previously mentioned genes in Cameroon were carried out in Yaoundé, which is not representative of the entire Central region in Cameroon. The drug resistance profile in Makenene, Central Cameroon, where the prevalence of malaria ranged from 40 % to 70 % between 2016 and 2021 (Djoufounna et al., 2022b, 2024), is still unknown. Moreover, there is no new data on the prevalence of molecular markers associated with antimalarial drug resistance in *P. falciparum* parasites since the COVID-19 pandemic in 2021 in Cameroon, with the exception of one study in the Central and North regions (Nkemngo et al., 2023). The present study aims to assess the prevalence and evolution of *P. falciparum* mutations in the *Pfcrt, Pfmdr1, Pfdhfr*, *Pfdhps,* and *PfK13* genes in Makenene.

## 2 Materials and methods

### 2.1 Study area

**Fig. 1:**
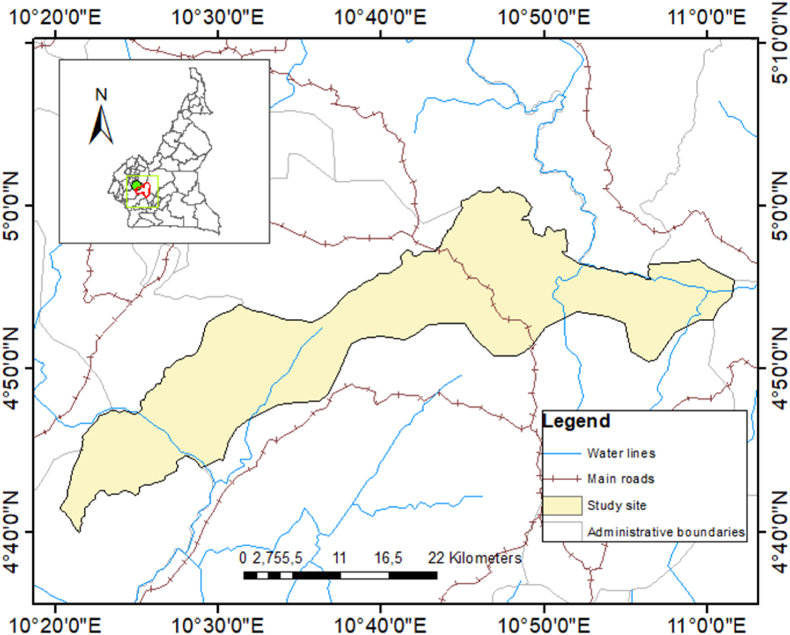
Map of the study site in Cameroon.

Makenene (4°53′04‴N; 10°47′40‴E) is a commune situated in the Mbam-et-Inoubou department in the Central Region of Cameroon (Fig. 1). Makenene is located at an elevation of 700 m above sea level and covers an area of 3500 square kilometres. The topography consists of two sets of high- and low-altitude plateaux. The climate is equatorial with two rainy seasons interspersed with two dry seasons, and temperatures stabilise around 25 °C. The first, shorter rainy season occurs from March to June, while the second, longer rainy season lasts from August to November. The long dry season runs from November to February, and the shorter one from June to August. Makenene is traversed by numerous rivers that form part of the Wouri and Sanaga basins. The natural climax vegetation in Makenene is dense evergreen Atlantic forest. Current non-forest areas are a result of human activity. In recent years, there has been an increase in migration due to the sociopolitical crisis in North-West and South-West regions. The primary economic activities are farming, trading, and breeding (Djoufounna et al., 2022a). Despite mosquito net distribution campaigns in the area, the prevalence of malaria caused by *P. falciparum* remains high (Djoufounna et al., 2022b, 2024).

### 2.2 Sample collection

A retrospective analysis was performed on the 406 samples collected between September and October 2021 as part of a previously described community-based cross-sectional study (Djoufounna et al., 2022b). In this previous study, the samples were stored at −20 °C for subsequent molecular analysis.

### 2.3 Automated extraction of malarial DNA using Qiacube

Genomic DNA was extracted from blood samples using the QIAamp 96 DNA Qiacube HT kit according to the manufacturer's recommendations (Qiagen GmbH, Germany).

### 2.4 Real time PCR for P. falciparum diagnosis

Molecular malaria diagnosis was performed using real time PCR on Light Cycler 2.0 (Roche Group, Switzerland) to identify *Plasmodium falciparum* species, as previously described (Wurtz et al., 2011). Briefly, *P. falciparum* was detected by individual real time PCR amplifications targeting specific genes using the Light Cycler® TaqMan® Master Mix (Roche Group, Switzerland). For each PCR run, two negative controls (water and human DNA) and a positive control (DNA from *P. falciparum*) were used.

### 2.5 Single nucleotide polymorphisms (SNPs) associated with antimalarial resistance in Pfcrt, Pfmdr1, Pfdhfr, Pfdhps, and PfK13

The analysis of *P. falciparum* polymorphisms in five genes, namely *Pfcrt, Pfmdr1, Pfdhfr, Pfdhps,* and *PfK13*, was conducted using PCR with the reaction conditions specified in Table 1. The reaction mixture consisted of 5 μL of genomic DNA, 1X PCR buffer, a 200 μM mixture of deoxynucleoside triphosphates (dGTP, dATP, dTTP, and dCTP) from Euromedex (Souffelweyersheim, France), variable concentrations of MgCl_2_ (as shown in Table 1), 0.32 μM of forward and reverse primers, and one unit of GoTaq G2 Flexi® DNA Polymerase from Promega (France), in a final volume of 25 μL. The thermal cycler was programmed as follows: initial denaturation at 95 °C for 5 min, followed by 35 cycles of denaturation at 95 °C for 30 s, specific hybridisation at a temperature indicated in Table 1 for variable elongation times, and extension at 72 °C for 1 min per 1000 base pairs. A final extension step at 72 °C for 5 min concluded the amplification. Purified genomic DNA from the *P. falciparum* clone 3D7 served as a positive control, while water and human DNA were used as negative controls. Sanger sequencing of the PCR products was performed using the primers described in Table 1. The obtained sequence data were analysed using Geneious Prime software v2.1 from Biomatters (New Zealand) to identify single nucleotide polymorphisms (SNPs) associated with antimalarial resistance genes (Table 2). The 3D7 (isolated in West Africa, obtained from MR4, Manassas, VA, USA), W2 (isolated in Indochina, obtained from MR4) and IPC 6261 (isolated in Cambodia, obtained from MR4) strains were used as control for sequencing.

**Table 1:** Primer sequences and PCR conditions for antimalarial resistance genes.

Gene	Primer Name	Primer Sequence	[MgCl_2_]	Tm
*Pfcrt*	CRT76-F	TTG GTA AAT GTG CTC ATG TG T	4.5 mM	55 °C
	CRT76-R	ACA AAT AAA GTT GTG AGT TTC GGA TG		
*Pfmdr1*-*1*	MDR1-1F	AGA GAA AAA AGA TGG TAA CCT CAG	2.5 mM	52 °C
	MDR1-1R	ACC ACA AAC ATA AAT TAA CGG		
*Pfmdr1*-*2*	MDR1-2F	CAG GAA GCA TTT TAT AAT ATG CAT	2.5 mM	56 °C
	MDR1-2R	CGT TTA ACA TCT TCC AAT GTT GCA		
*Pfdhfr*	Dhfr-F	ACG TTT TCG ATA TTT ATG C	2.5 mM	52 °C
	Dhfr-R	TCA CAT TCA TAT GTA CTA TTT ATT C		
*Pfdhps*	Dhps-F	AAC CTA AAC GTG CTG TTC AA	2.5 mM	59 °C
	Dhps-R	TCC AAT TGT GTG ATT TGT CCA CAA		
*PfK13*	*K13-F*	GGG AAT CTG GTG GTA ACA GC	2.5 mM	58 °C
	*K13-R*	CGG AGT GAC CAA ATC TGG GA		
*PfK13 nested*	*nestK13-F*	GCC TTG TTG AAA GAA GCA GA	2.5 mM	60 °C
	*nestK13-R*	GCC AAG CTG CCA TTC ATT TG		

F: Forward primer, R: Reverse primer.

**Table 2:** Single nucleotide polymorphisms associated with antimalarial resistance genes.

Gene		Wild-type		Mutant	
	Codon (AA)	SNP	AA	SNP	AA
*Pfdhfr*	153 (51)	aat	N	a**t**t	I
	177 (59)	tgt	C	**c**gt	R
	324 (108)	agc	S	a**a**c	N
	492 (164)	ata	I	**t**ta	L
*Pfdhps*	1293 (431)	ata	I	**g**ta	V
	1308 (436)	tct	S	T**t**t/**g**ct	F/A
	1311 (437)	gct	A	g**g**t	G
	1620 (540)	aaa	K	**g**aa	E
	1743 (581)	gcg	A	g**g**g	G
	1839 (613)	gcc	A	**a**cc	S
*Pfcrt*	216 (72)	tgt	C	t**c**t	S
	219 (73)	gta	V	gt**c**	V
	222 (74)	atg	M	at**t**	I
	225 (75)	aat	N	**g**a**a**	E
	228 (76)	aaa	K	a**c**a	T
	1068 (356)	ata	I	a**c**a	T
*Pfmdr1*	258 (86)	aat	N	**t**at	Y
	552 (184)	tat	Y	t**t**t	F
	3102 (1034)	agt	S	**t**gt	C
	3126 (1042)	aat	N	**g**at	D
	3738 (1246)	gat	D	**t**at	Y
*PfK13*	1428 (476)	atg	M	at**a**	I
	1479 (493)	tac	Y	**t**ac	H
	1617 (539)	aga	R	a**c**a	T
	1629 (543)	att	T	a**c**t	T
	1722 (574)	cct	P	c**t**t	L
	1740 (580)	tgt	C	t**a**t	Y

AA: amino acid, SNP: single nucleotide polymorphism, mutated nucleotide in bold.

### 2.6 *Data management and statistical analysis*

Data were recorded in Microsoft Excel (Office, 2013) and analysed using the R software. BioEdit software was used to compare and align nucleotide sequences, aiding in the identification of genetic variations linked to drug resistance in malaria parasites.

## 3 Results

### 3.1 *Molecular malaria diagnosis*

Genomic material isolated from the 406 blood samples was analysed using qPCR techniques, revealing that 185 samples tested positive for *P. falciparum* parasites. This prevalence was higher than that obtained through previously reported rapid diagnostic tests and blood smear results in the community-based cross-sectional study (170) (Djoufounna et al., 2022b). The 185 positive samples were used to genotype *P. falciparum* resistance genes.

### 3.2 *Prevalence of genotypes in different resistance genes*

#### 3.2.1 Pfdhfr (dihydrofolate reductase)

Of the 121 successfully analysed samples for the *Pfdhfr* gene, 4 samples carried multiple *P. falciparum* infection (2 mixed alleles N51 + 51I and 2 mixed alleles C59 + 59R) (Table 3). One hundred percent of the samples harboured parasites with the **IRN**I haplotype (triple mutation of codons 51, 59, and 108 and wild-type for codon 164) (Table 3). The haplotypes **I**C**N**I and N**RN**I were observed in 1.7 % of the isolates.

**Table 3:** Frequency of *Pfdhfr* genotypes in *P. falciparum* isolates from Makenene.

Haplotypes				Proportions	Percentage
N51I	C59R	S108N	I164L	(n/N)	(%)
I	R	N	I	117/121	96.6
I	C/R	N	I	2/121	1.7
N/I	R	N	I	2/121	1.7

n: number of cases, N: total.

#### 3.2.2 Pfdhps (dihydropteroate synthase)

Prevalence of the different mutations of the *Pfdhps* gene are shown in Table 4. In total, the mutation from serine to alanine at position 436 (S436A) was found with a prevalence of 67.6 %. The A437G mutation (alanine to glycine) at position 437 was present in 90.7 % of the samples (98/108). The A581G mutation (alanine to glycine) at position 581 was found in 44.4 % of the isolates. The mutation from alanine to serine at position 613 (A613S) was observed in 46.3 % (50/108) of the samples. The prevalence of the K540E mutation was found in only 1.9 % of the cases.

**Table 4:** Frequency of *Pfdhps* genotypes in *P. falciparum* isolates from Makenene.

Haplotypes						Proportions	Percentage
I431V	S436A	A437G	K540E	A581G	A613S	(n/N)	(%)
I	S	A	K	A	A	1/108	0.9
I	**S**	**G**	K	A	A	23/108	21.3
ND	**S**	**G**	K	A	A	8/108	7.4
I	**S**	**G**	K	A/**G**	A	1/108	0.9
I	**S**	**G**	K/**E**	A	A	1/108	0.9
ND	**S**	**G**	K/**E**	A	A	1/108	0.9
I	**A**	A	K	A	A	8/108	7.4
ND	**A**	A	K	A	A	1/108	0.9
I	**A**	A/**G**	K	A	A	1/108	0.9
ND	**A**	A/**G**	K	A	A	1/108	0.9
I	**A**	G	K	A	A	1/108	0.9
**V**	**A**	G	K	A	A	1/108	0.9
ND	**A**	G	K	A	A	6/108	5.6
I	**A**	A/**G**	K	**G**	**S**	3/108	2.8
I/V	**A**	A/**G**	K	**G**	**S**	1/108	0.9
ND	**A**	A/**G**	K	A/**G**	A/S	1/108	0.9
I	**A**	**G**	K	**G**	**S**	2/108	1.9
**V**	**A**	**G**	K	**G**	**S**	9/108	8.3
ND	**A**	**G**	K	**G**	**S**	2/108	1.9
I	S/**A**	**G**	K	A	A	2/108	1.9
ND	S/**A**	**G**	K	A	A	1/108	0.9
ND	S/**A**	**G**	K	A	**S**	1/108	0.9
ND	S/**A**	**G**	K	A	A/**S**	1/108	0.9
I/**V**	S/**A**	**G**	K	**G**	A/**S**	1/108	0.9
I/**V**	S/**A**	**G**	K	A/**G**	A/**S**	1/108	0.9
I	S/**A**	**G**	K	A/**G**	A/**S**	11/108	10.2
ND	S/**A**	**G**	K	A/**G**	A/**S**	16/108	14.8
ND	S/**A**	A/**G**	K	A	A	1/108	0.9
I	S/**A**	A/**G**	K	A	A/**S**	1/108	0.9

ND: not determined.

The new I431V mutation was observed in 18.8 % (13/69) of the samples with 12 isolates (17.4 %) with parasites harbouring this mutation, associated with mutations on codons 436, 437, 581, and 613.

At least 43 % (46/108) of samples carried multiple *P. falciparum* infection. Fifty *Pfdhps* haplotypes (codons 436, 437, 540, 581, and 613) were observed: S**G**KAA (simple mutation, 68/108, 63.0 %), **AG**K**GS** (quadruple mutation, 67/108, 62.0 %), **AG**KAA (double mutation, 41/108, 38.0 %), S**G**KA**S** (double mutation, 33/108, 30.6 %), A**G**KA**S** (double mutation, 31/108, 28.7 %), S**G**K**G**A (double mutation, 30/108, 27.8 %), S**G**K**GS** (triple mutation, 29/108, 26.9 %), A**G**K**G**A (double mutation, 29/108, 26.9 %), **A**AKAA (simple mutation, 15/108, 13.9 %), **A**AK**GS** (triple mutation, 5/108, 4.6 %), **A**AKA**S** (double mutation, 3/108, 2.8 %), SAKAA (wild-type, 3/108, 2.8 %), **A**AK**G**A (double mutation, 2/108, 1.9 %), S**GE**AA (double mutation, 2/108, 1.9 %), and SAKA**S** (simple mutation, 1/108, 0.9 %).

The septuple mutant parasites (**IRN**I + **AG**K**GS** haplotypes) were detected in 62.0 % of the isolates.

#### 3.2.3 Pfcrt (chloroquine resistance transporter)

Concerning the chloroquine resistance transporter (CRT), the haplotype for the 5 codons (72, 73, 74, 75, 76, 356) was analysed for only 108 samples. The CQ-susceptible wild-type haplotype CVMNK (codon 72–76) was identified in 100 % of the interpretable isolates (108), while the K356T mutation (isoleucine to threonine) was detected in only 2.3 % (3/128) of the samples (Table 5).

**Table 5:** Frequency of *Pfcrt* genotypes in *P. falciparum* isolates from Makenene.

Haplotypes						Proportions	Percentage
C72S	V73V	M74I	N75E	K76T	I356T	(n/N)	(%)
ND	ND	ND	ND	ND	I	19/128	14.8
ND	ND	ND	ND	ND	**T**	1/128	0.8
C	V	M	N	K	I	106/128	82.8
C	V	M	N	K	**T**	2/128	1.6

ND: not determined.

#### 3.2.4 Pfmdr1 (multidrug resistance protein 1)

Among the 157 cases successfully analysed for the five codons, 98.7 % (155/157) had the wild-type allele N86, while 184F was detected in 74.5 % of the isolates (117/157) (Table 6). All the samples were wild-type for codons S1034C, N1042D, and D1246Y.

**Table 6:** Frequency of *Pfmdr1* genotypes in *Plasmodium falciparum* isolates from Makenene.

N86Y	Y184F	S1034C	N1042D	D1246Y	(n/N)	(%)
ND	ND	S	N	D	9/9	100
N	F	S	N	D	96/157	61.1
N	Y	S	N	D	40/157	25.5
N	Y/F	S	N	D	16/157	10.2
N/Y	F	S	N	D	3/157	1.9
Y	F	S	N	D	2/157	1.3

ND: not determined.

The haplotypes N86/184F and 86Y/Y184 were observed in 73.2 % and 0 % of the isolates, respectively.

#### 3.2.5 PfK13 (kelch 13)

No previous reported mutations or new mutations were detected on the K13-propeler domain gene containing the 6 blades involved in artemisinin resistance (from codon 440 to 700) in the 155 evaluated isolates.

## 4 Discussion

Antimalarial drug resistance is a critical issue requiring a multifaceted approach to combat evolving resistance patterns. For better management and treatment of malaria cases, delineating the genetic profile of the main genes associated with malaria drug resistance used for chemotherapy in Cameroon is crucial. Genetic mutations and drug efflux mechanisms play pivotal roles in the development of resistance. Makenene's position as a crossroads and resting place for travellers, combined with the presence of internally displaced persons, can contribute to the presence of the mutant phenotypes. Makenene is located in an ecoepidemiological setting with a high prevalence of malaria (Djoufounna et al., 2022b, 2024) and a high variability and intensity in the use of antimalarial drugs with insufficient regulation (Niba et al., 2023). SP was adopted in Cameroon for the treatment of malaria over 30 years ago. SP resistance is determined by the presence of mutations in the *Pfdhfr* and *Pfdhps* genes. The S108N mutation in *Pfdhfr* is associated with in vivo and in vitro pyrimethamine resistance (Sibley et al., 2001; Picot et al., 2009). The addition of N51I, C59R, or I164L mutations increases the level of in vivo and in vitro resistance to pyrimethamine and SP (Peterson et al., 1988; Picot et al., 2009), while the S436A, A437G, and K540E mutations confer sulfadoxine resistance (Wang et al., 1997; Kublin et al., 2002; Lumb and Sharma, 2011). This study exhibited a very high proportion of the *Pfdhfr* **IRN**I haplotype (100 %). These results were consistent with those of previous studies on samples collected in Cameroon countrywide since 2016 (Wang et al., 2022), in Yaoundé (Tuedom et al., 2021; Sarah-Matio et al., 2022; Cohen et al., 2023; Nana et al., 2023; Niba et al., 2023; Nkemngo et al., 2023) and in the Far North (Guémas et al., 2023). At the same time as the collection of samples for our study in 2021, the prevalence of the **IRN**I haplotype in imported falciparum malaria from Cameroon to France was 93 % (CNR, 2024). The breadth of the polymorphism of the pyrimethamine-associated *Pfdhfr* marker revealed a complete fixation of the triple mutant **IRN**I.

The *Pfdhps* A437G mutation, associated with partial resistance to sulfadoxine, was detected at high prevalence (90.7 %), consistent with previous studies (Tuedom et al., 2021; Sarah-Matio et al., 2022; Wang et al., 2022; Cohen et al., 2023; Guémas et al., 2023; Nana et al., 2023; Niba et al., 2023; Nkemngo et al., 2023). The prevalence of the *Pfdhps* A613S mutation (46.3 %) was higher than that observed in parasites collected from 2016 to 2021 in other areas of Cameroon (0 %–11 %) (L'Espiscopia et al., 2021; Sarah-Matio et al., 2022; Wang et al., 2022; Cohen et al., 2023; Guémas et al., 2023; Nana et al., 2023; Niba et al., 2023). Regarding the *Pfdhps* A581G mutation, its significant involvement in sulfadoxine resistance is widely recognised. Indeed, the identification of the super resistant mutations *Pfdhps* A581G (44.4 %) and A613S (46.3 %) in Makenene highlights their substantial contribution to the increase in resistance levels. In the majority of the studies, the prevalence of the A581G mutation was below 10 % (Tuedom et al., 2021; Sarah-Matio et al., 2022; Wang et al., 2022; Guémas et al., 2023; Nana et al., 2023; Niba et al., 2023; Xu et al., 2023) except in Yaoundé in 2020 (24.3 %) (Cohen et al., 2023), in Mont Cameroon in 2016 and 2017 (52.4 %) (Mbacham et al., 2023), and in Maroua in 2017 (Guémas et al., 2023).

The *Pfdhps* K540E mutation, linked to “super” or full resistance to SP when associated with N51I, N59R and S108N mutations in *Pfdhfr* and A437G in *Pfdhps* (Picot et al., 2009; Naidoo and Roper, 2013; Mita et al., 2014), was detected in only two isolates (1.9 %). This result was consistent with previous studies in Tibati, Mfou, Maroua, Mount Cameroon, and Yaoundé, where this genetic variant was observed in only a small number of isolates (Tuedom et al., 2021; Sarah-Matio et al., 2022; Cohen et al., 2023; Guémas et al., 2023; Mbacham et al., 2023; Nana et al., 2023; Niba et al., 2023). In East Africa, the widespread prevalence of the *Pfdhps* K540E mutation is well-documented, indicating its common occurrence with rates over 90 % (Kavishe et al., 2016; Gikunju et al., 2020).

The septuple mutant parasites (**IRN**I + **AG**K**GS** haplotypes) were detected in 62.0 % (67/108) of the isolates. The quadruple **AG**K**GS** haplotype was found at 8.6 % of isolates in Dschang in 2017 (L'Espiscopia et al., 2021) and the **IRN**I + **AG**K**GS** haplotypes at 2.8 % Yaoundé (Nana et al., 2023) and 2.9 % in Douala in 2015–2016 (Eboumbou Moukoko et al., 2023). The presence of these septuple mutant parasites in high proportions, associated with high levels of *P. falciparum* complete resistance to SP, suggests a decrease in the efficacy of intermittent preventive treatment. The effectiveness of chemoprevention and intermittent preventive treatment is currently being evaluated in several studies in Cameroon (Martinez-Vega et al., 2024; Stresman et al., 2024).

The new I431V mutation, which is overrepresented in patients taking SP, was observed in 18.8 % (13/69) of the samples. This mutation had already been detected in Cameroon in 2010–2011 (9.8 %) (Chauvin et al., 2015) and in 2020–2021 (25.7–32.3 %) (Nkemngo et al., 2022; Cohen et al., 2023). Moreover, 17.4 % of the falciparum isolates (12/69) harboured the **VAG**K**GS** haplotype. This haplotype ranged between 4 % in Tibati to 48.6 % in Maroua (Guémas et al., 2023). The octuple mutant (**IRN**I + **VAG**K**GS**) (17.4 %) was also detected in 2010 and 2020 in Yaoundé (Chauvin et al., 2015; Cohen et al., 2023) and in imported falciparum malaria from Cameroon to China collected between 2021 and 2024 (5 %) (Wang et al., 2022). This haplotype was overrepresented in parasites sampled from pregnant women who were taking intermittent preventive treatment with SP (3.4 % versus 21.4 %) (Chauvin et al., 2015).

The *Pfcrt* K76T mutation and the CIEVT haplotype (codon 72 to 76) are strongly associated with in vitro and in vivo resistance to chloroquine (Wootton et al., 2002; Johnson et al., 2004; Picot et al., 2009). The CQ-susceptible wild-type haplotype *Pfcrt* CVMNK (codon 72–76) was detected in 100 % of the samples. This absence or low prevalence of CVIET was consistent with recent data from falciparum malaria imported from Cameroon to France collected between 2021 and 2024 (3 % in 2021, 1 % in 2022, 2 % in 2023, and 5 % in 2024) (CNR, 2024) and with isolates collected countrywide in Cameroon between 2013 and 2021 (Foguim et al., 2020; Wang et al., 2020; L'Espiscopia et al., 2021; Tuedom et al., 2021; Sarah-Matio et al., 2022; Niba et al., 2023). Nonetheless, the prevalence of these susceptible parasites varies across different localities, possibly influenced by the diverse utilisation of chloroquine within each community. The prevalence of the mutant CVIET haplotype has decreased significantly over the last 20 years: >80 % before 2006 (Apinjoh et al., 2017; Ndam et al., 2017; Niba et al., 2021; Moyeh et al., 2022), between 23 % and 70 % from 2007 to 2016 (Mbenda and Das, 2014; Apinjoh et al., 2017; Ndam et al., 2017; Niba et al., 2021, 2023; Moyeh et al., 2022), and below 25 % since 2019 (Ali et al., 2022; Moyeh et al., 2022; Sarah-Matio et al., 2022; Niba et al., 2023; Xu et al., 2023; CNR, 2024). In reality, the removal of CQ drug pressure has resulted in the reemergence of CQ-susceptible parasites.

The *Pfcrt* I356T mutation, associated with artemisinin resistance in South East Asia (Miotto et al., 2015), was observed in only two isolates (1.9 %). A prevalence of 56.1 % was previously found in falciparum samples from falciparum malaria imported from Cameroon to France and collected between 2017 and 2018 (Foguim et al., 2020). This mutation was mainly observed in the Côte d’Ivoire (Foguim et al., 2020), in the Gambia, and in isolates from Thailand and Cambodia (Dhingra et al., 2019).

With regards to the *Pfmdr1* gene, the wild-type N86 allele, which is significantly reported in parasites from a recrudescence in patients treated with AL (Sosowath et al., 2005; Dokomajilar et al., 2006; Venkatesan et al., 2014; Baraka et al., 2015; Sondo et al., 2016), was observed in 98.7 % of the isolates studied in 2021 in Makenene. A high prevalence (>60 %) has also been demonstrated in other areas of Cameroon since 2019 (L'Espiscopia et al., 2021; Niba et al., 2021, 2023; Ali et al., 2022; Moyeh et al., 2022; Sofeu-Feugaing et al., 2023; Xu et al., 2023). The prevalence of the wild-type N86 has increased significantly over the last 20 years, appearing in less than 20 % of samples before 2009 (Menard et al., 2012; Niba et al., 2021; Moyeh et al., 2022). In contrast, the 86Y mutation, which modulates in vitro susceptibility to most of the artemisinin partners including artemisinin, amodiaquine, mefloquine, lumefantrine, and piperaquine (Wootton et al., 2002; Sidhu et al., 2005; Veiga et al., 2016; Gendrot et al., 2019) and is selected in DHPQ or ASAQ recurrent infections (Venkatesan et al., 2014; Baraka et al., 2015; Nankabirwa et al., 2016; Sondo et al., 2016; Taylor et al., 2016; Yeka et al., 2016), was detected at a low prevalence (1.3 %). The Y184F mutation was observed in 74.5 % of the samples. These data were consistent with data from 2019 (L'Espiscopia et al., 2021; Niba et al., 2023; Sofeu-Feugaing et al., 2023). The N86/184F (NF) haplotype, selected in parasites with a recrudescence in patients treated with AL (Sosowath et al., 2005; Dokomajilar et al., 2006; Venkatesan et al., 2014; Baraka et al., 2015; Sondo et al., 2016), was found in 73.2 % of the samples. This prevalence was consistent with those (>40 %) of falciparum malaria imported from Cameroon to France (collected since 2021) (CNR, 2024) and China (collected between 2016 and 2021) (Xu et al., 2023), and collected in Dschang in 2017 (L'Espiscopia et al., 2021). A study in Yaoundé in 2019–2020 showed a low prevalence (3 %) for the NF haplotype (Niba et al., 2023).

No isolate harboured the 86Y/Y184 (YY) haplotype, selected in parasites with a recrudescence in patients treated with DHPQ (Nankabirwa et al., 2016; Taylor et al., 2016). This result was consistent with the low prevalence of the YY haplotype observed in falciparum malaria imported from Cameroon to France collected since 2021 (0–3 %) (CNR, 2024) and China collected from 2016 to 2021 (3.5 %) (Xu et al., 2023), and collected in Dschang in 2017 (0.9 %) (L'Espiscopia et al., 2021) and in Yaoundé in 2014 before the adoption of artemisinin-based therapy (ACT) and in 2019–2020 after the adoption of ACT (0.6 % and 0.5 %, respectively (Niba et al., 2023). This YY haplotype was essentially found in parasites from the Union of Comoros (21 % in 2021 to 43 % in 2024) (CNR, 2024).

All parasites in this study also carried the wild-type allele D1246. The studies carried out in Cameroon since 2018 showed the prevalence of the D1246Y mutant ranged from 0 % in Douala to 48 % in Mount Cameroon (Tuedom et al., 2021; Moyeh et al., 2022; Sofeu-Feugaing et al., 2023). The NFD (N86/184F/D1246) haplotype was detected at a frequency of 62.1 % in the present study in Makenene versus 26.6 % in Yaoundé in 2019–2020 (Niba et al., 2023) and 37 % in Dschang in 2017 (L'Espiscopia et al., 2021).

Artemisinin resistance was initially identified in Cambodia (Noedl et al., 2008; Dondorp et al., 2009, WHO, 2018) and spread rapidly throughout South East Asia (Phyo et al., 2012; Ashley et al., 2014; Imwong et al., 2017). In vivo resistance to artemisinin derivatives remains relatively rare but partial resistance resulting in a delayed parasite clearance has emerged in eastern Africa (Uwimana et al., 2021; Straimer et al., 2022; Angwe et al., 2024; Ishengoma et al., 2024). In 2014, different mutations in *PfK13* were shown to be associated with partial resistance to artemisinin derivatives in Asia (F446I, N458Y, M476I, Y493H, R539T, I543T, P553L, P574L, and C580Y) (Ariey et al., 2014). These mutations, and more particularly the C580Y mutation, have been not yet reported in Africa but new mutations (C469Y, R561H, R622I, and A675V) have been identified in Africa and are associated with increased in vitro parasite survival rates and with delayed parasite clearance (Delandre et al., 2024). No previous reported mutation or new mutation was detected on the K13-propeler domain gene in the *P. falciparum* collected in Makenene. None of the mutations associated with the emergence of partial resistance to artemisinin in Africa (C469Y, R561H, R622I and A675V) has been reported in Makenene or in countrywide Cameroon (Menard et al., 2016; Djaman et al., 2017; Eboumbou Moukoko et al., 2019; Wang et al., 2020; Mairet-Khedim et al., 2021; Nkemngo et al., 2023; Kojom Foko et al., 2025; Milong Melong et al., 2025). The last published study on ACT effectiveness took place in Yaoundé in 2019–2020 and showed that the two combinations of artesunate-amodiaquine and artemether-lumefantrine were still effective. However, no new data have been available since 2020.

One of the limitations of this work is the lack of evaluation of the number of copies of *pfmdr1*, which is involved in resistance to mefloquine (Barnes et al., 1992; Price et al., 1999).

The findings underscore the urgent need for continued surveillance and monitoring of drug resistance patterns to guide effective malaria control strategies in the area.

## 5 Conclusion

The in-depth analysis of genetic mutations associated with antimalarial drug resistance in this study, notably with the high prevalence of mutations on the *Pfdhfr* and *Pfdhps* genes, highlights the immediate need for proactive strategies to combat the evolving resistance in Makenene. None of the mutations associated with the emergence of partial resistance to artemisinin in Africa or in South East Asia were detected. Continuous monitoring, including molecular surveillance as well as in vivo surveillance is crucial to uphold the effectiveness of current antimalarial treatments and, more particularly ACTs, and to enable a better decision-making around effective treatment policies in Cameroon and in Africa as a whole.

## CRediT authorship contribution statement

**Nelly Armanda Kala-Chouakeu:** Writing – original draft, Investigation, Formal analysis, Data curation. **Joel Djoufounna:** Resources, Investigation. **Nicolas Benoit:** Validation, Resources, Investigation, Formal analysis, Data curation. **Timoléon Tchuinkam:** Writing – review & editing, Supervision, Methodology, Conceptualization. **Océane Delandre:** Writing – review & editing, Resources, Investigation, Formal analysis. **Lionel Almeras:** Resources, Methodology. **Roland Bamou:** Writing – review & editing, Resources, Investigation. **Christophe Antonio-Nkondjio:** Supervision, Resources, Conceptualization. **Marylin Madamet:** Writing – review & editing, Supervision, Resources, Methodology, Formal analysis. **Bruno Pradines:** Writing – review & editing, Writing – original draft, Validation, Supervision, Resources, Project administration, Funding acquisition, Formal analysis, Conceptualization.

## Data availability statement

The datasets used and analysed in this study are available from the corresponding author on reasonable request.

## Ethical statement

Before the study began, authorisation was obtained from the Director of the District Medical Centre (DMC) of Makenene, and an administrative permit was secured from the Divisional Officer of the Makenene district (No. 04/AR/JOR-04/SP). Additionally, the study received approval from the Regional Committee for Ethics and Human Health Research of the Centre Region (CE No. 1289/CRERSHC/2021). Verbal consent was first obtained from the heads of households, followed by written consent from each participant before their inclusion in the study. Participants were informed about the confidentiality and voluntary nature of the study and assured they could withdraw at any time.

## Funding

This work was supported by the Direction Générale de l’Armement [grant NBC2-B-2120], by the National Research Agency under the “Investments for Future” programme [grant 10.13039/501100001665ANR10-IAHU] and by the European Regional Development Fund (10.13039/501100002924FEDER) [grant 10.13039/501100002924FEDER IHUPERF].

## Declaration of competing interest

The authors declare that they have no competing interests.
